# Cognitive restraint, uncontrolled eating, and emotional eating. The Italian version of the Three Factor Eating Questionnaire-Revised 18 (TFEQ-R-18): a three-step validation study

**DOI:** 10.1007/s40519-024-01642-y

**Published:** 2024-02-24

**Authors:** Alessandro Alberto Rossi, Giada Pietrabissa, Gianluca Castelnuovo, Stefania Mannarini

**Affiliations:** 1 Department of Philosophy, Sociology, Education, and Applied Psychology, Section of Applied Psychology, University of Padova, Padua, Italy; 2 Center for Intervention and Research on Family studies – CIRF, Department FISPPA, University of Padova, Padua, Italy; 3 Clinical Psychology Research Laboratory, IRCCS Istituto Auxologico Italiano, Milan, Italy; 4 Dipartimento di Psicologia, Università Cattolica del Sacro Cuore, Milan, Italy

**Keywords:** Eating behavior, Three-Factor Eating Questionnaire, Eating disorders, Cognitive restraint, Uncontrolled eating, Emotional eating, Exploratory graph analysis, Confirmatory factor analysis

## Abstract

**Background:**

The Three Factor Eating Questionnaire-Revised 18 (TFEQ-R-18) is an extensively used questionnaire to measure three transdiagnostic features of eating behavior: cognitive restraint, uncontrolled eating, and emotional eating.

**Objective:**

This research aims to investigate the psychometric properties of the Italian version of the TFEQ-R-18 in three large community samples.

**Method:**

Cross-sectional research designs were employed. In Study 1 (*N* = 537), an exploratory graph analysis (EGA) was used to examine item clustering within the TFEQ-R-18. In Study 2 (*N* = 645), a confirmatory factor analysis (CFA) was conducted to test its structural validity. In Study 3 (*N* = 346), a MANOVA was employed assessing mean differences across eating disorders (e.g., anorexia nervosa, bulimia nervosa, binge eating disorder).

**Results:**

In Study 1, the EGA accurately identified the three original dimensions of the TFEQ-R-18. Study 2 showed that the Italian TFEQ-R-18 has good fit indexes (CFI = 0.989, RMSEA = 0.064; 90% CI [0.058, 0.070], SRMR = 0.062), and possesses robust psychometric properties. Study 3 reveals distinct, statistically significant differences among eating disorders.

**Conclusion:**

The TFEQ-R-18 proves to be a concise and precise tool for measuring transdiagnostic eating behaviors. Its applicability in the Italian context, supported by robust psychometric properties, suggests its utility for both research and clinical purposes. The findings affirm its potential to inform interventions aimed at enhancing psychological health.

**Level of evidence:**

Level V, descriptive study.

**Supplementary Information:**

The online version contains supplementary material available at 10.1007/s40519-024-01642-y.

## Introduction

Feeding and Eating disorders (FEDs) are serious pathologies characterized by dysfunctional eating behaviors that have a severe negative impact on the physical health and psychosocial functioning of the person [1, 2]. International epidemiological research indicated a lifetime prevalence of EDs in Western countries as follows: anorexia nervosa (AN) at 0.16%; bulimia nervosa (BN) at 0.63%; and Binge Eating Disorder (BED) at 1.53% [3].

Dysfunctional eating behaviors commonly develop from “dieting or restrained eating” behavior to pursue an ideal of thinness and to be more attractive to the eyes of modern society [4, 5]. If dieting continues, a vicious circle of dysfunctional eating behaviors progresses with both psychological and physiological negative reinforcement (i.e., reduction of discomfort) [2, 4, 5]. The restraint theory investigates how the dimension of “Cognitive Restraint”—efforts and worries to regulate food intake to control body weight and shape—may cause several and recurrent episodes of overeating, up to the potential development of obesity and eating disorders [6]. Many studies have linked (cognitive) restraint to binge eating behaviors and weight gain, while other studies found an association between higher levels of restraint and lower BMI [7].

Another essential aspect of dysfunctional eating behavior is “Uncontrolled Eating”, defined as the tendency to lose control and overeat. Despite overeating being normal when confined to some particular occasions, it may start to become dysfunctional when it occurs regularly, despite the individual efforts to resist and the several attempts to make healthier food choices [8]. Individuals with several episodes of overeating report the continuous feeling of being out of control and this sensation is also common to patients with various eating disorders, such as BN and BED, as well as obesity [9]—that do to date affects more than 600 million people worldwide [10], and it is associated with several physical and psychological conditions [11].

A third component/aspect characterizing dysfunctional eating patterns is “Emotional Eating” defined as the tendency to eat in response to emotional urges, both positive and negative such as distress and negative emotions [12, 13]. That may lead to compulsive/emotional eating [8, 14, 15]. Emotional eating has an important clinical significance because patients with eating disorders often associate binge eating episodes with negative affective states [12, 16, 17]—and it seems to be an antecedent of binge eating episodes [12, 16].

It appears evident how these three components/aspects are implicated in all eating disorders in a transdiagnostic way, and should be carefully investigated for comprehensive evaluation of eating behaviors [2, 6, 7, 9, 16, 18, 19].

In this scenario, a better understanding of eating behaviors—in their cognitive, behavioral, and emotional aspects—is essential for clinicians and researchers to delivery prevention programs and structured support [4, 20]. Consequently, a brief, accurate but comprehensive measurement tool is needed.

One of the most widely used measures in the field of eating behavior research is the Three Factor Eating Questionnaire, TFEQ, developed by Stunkard and Messick [13, 21]. The original TFEQ contained 51 items divided in two different parts/sections and designed to assess three cognitive and behavioral domains (or ‘factors’) of eating: “Cognitive Restraint” (CR), “Uncontrolled Eating” (UE), and “Emotional Eating” (EE). Also, the 51 item of the TFEQ have been translated in many languages—such as English, French, German, Dutch, Spanish, Turkish, and Finnish [4, 6, 21–26].

However, despite its large use across different countries, contexts and populations, several studies have raised concerns about its factor structure and factors stability [6, 13, 27].

Therefore, a revised version of the questionnaire (namely, the TFEQ-R-18) was created taking into consideration the three above-mentioned key dimensions of eating behavior: “Cognitive Restraint” (CR), “Uncontrolled Eating” (UE), and “Emotional Eating” (EE) [13, 28]. Using the most efficient items to boost the convergent and discriminant validity of the new scales, a revised, shorter version of the questionnaire was created [13, 28]. Internal consistency estimates (Cronbach's alpha) were above the 0.70 standard and tests of the internal structure of the instrument were satisfactory [6]. Moreover, the TFEQ-R-18 seems to maintain adequate (or even better) convergent and discriminant validity than the original 51-item version [26, 29]. In addition, given its brevity and ease of administration, it represents a practical tool for clinical practice and research [26, 29]—considering the cross-cultural diffusion as well.

However, to date, the Italian validation of the TFEQ-R-18 version is still lacking. Thus, providing the Italian validation of the TFEQ-R-18 represents a step forward in the field of measurement applied to (dysfunctional) eating behaviors and it is of primary importance to provide a proper validation and investigation of its properties in the Italian context.

Therefore, the present three-step study aims to assess the psychometric properties of the Italian version of the TFEQ-R-18 in three large community samples. More in detail, an exploratory graph analysis (EGA) was employed to examine the TFEQ-R-18 item clustering (Study 1). Following, confirmatory factor analysis (CFA) was run for a deep investigation of the psychometric properties of the questionnaire (Study 2). Lastly, an analysis of mean differences (MANOVA) was conducted among various diagnostic clusters (Study 3).

## Study 1. The dimensional structure of the Italian TFEQ-R-18

### Methods and materials

#### Translation and cultural adaptation

Guidelines for questionnaires translation process were followed [30]. Two experienced clinical psychologists independently translated the TFEQ-R-18 from English to Italian and an independent translator performed a back-translation. The TFEQ-R-18 was then tested on a sample of 20 individuals to assess the understandability of the items. No further modifications were necessary. The final version is reported in the supplementary materials (Supplementary 1).

#### Sample size determination

The sample size was selected a priori. A sample of at least 500 individuals was considered adequate to perform the main statistical analysis of the study [31].

#### Procedure

Following the methodology of prior studies [8, 14, 15, 32] participants from the general population were enrolled via social media platforms (i.e., Facebook, Twitter, Instagram, etc.) [33]. The study received ethical approval from the Ethics Committee of the IRCCS Istituto Auxologico Italiano - protocol number 2020_02_18_04.

Inclusion criteria were: (A) being aged over 18 years, (B) being a native Italian speaker. Exclusion criteria were: (C) uncomplete assessment procedure (i.e., missing answers) and (D) not providing the informed consent to participate in the study.

#### Participants

The sample consisted of 537 participants: 136 males (25.3%) and 401 females (74.7%), aged between 18 and 83 years (mean = 36.39, SD = 15.148), and with a BMI ranging from 15.79 to 47.35 kg/m^2^ (mean = 23.147, SD = 4.133)—Table 1 provides a complete description of the sample.

**Table 1:** Study 1, Study 2, and Study 3. Samples descriptive statistics

	Study 1 (*N* = 537)		Study 2 (*N* = 645)		Study 3 (*N* = 346)	
Age (mean. *SD*)	36.39	15.148	33.18	15.823	36.23	14.512
BMI (mean. *SD*)	23.15	4.133	23.44	5.421	24.88	6.328
Gender (*n. %*)						
Male	136	25.3%	171	26.5%	56	16.2%
Female	401	74.7%	474	73.5%	290	83.8%
Civil status (*n. %*)						
Single	152	28.3%	202	31.3%	101	29.2%
In a relationship	194	36.1%	295	45.7%	126	36.4%
Married	156	29.1%	126	19.5%	93	26.9%
Separated/divorced	28	5.2%	10	1.6%	22	6.4%
Widowed	7	1.3%	12	1.9%	4	1.2%
Education (*n. %*)						
Middle school degree	33	6.1%	61	9.4%	23	6.7%
High school degree	218	40.6%	248	38.4%	136	39.3%
Bachelor degree	253	47.1%	313	48.5%	161	46.5%
Master/Ph.D	33	6.1%	23	3.6%	26	7.5%
Work status (*n. %*)						
Student	191	35.6%	190	29.5%	119	34.4%
Dependent worker	238	44.3%	255	39.5%	163	47.1%
Entrepreneurs/freelancers	52	9.7%	113	17.5%	27	7.8%
Housewife	5	0.9%	4	0.6%	5	1.4%
Unemployed	17	3.2%	33	5.1%	16	4.6%
Retired	34	6.3%	50	7.8%	16	4.6%
BMI class (*n. %*)						
Severely underweight (< 16)	3	0.6%	11	1.7%	6	1.7%
Underweight (16–18.49)	38	7.1%	78	12.1%	19	5.5%
Normal weight (18.5–24.99)	361	67.2%	382	59.2%	197	56.9%
Overweight (25–29.99)	98	18.2%	64	9.9%	74	21.4%
Class I obesity (30–34.99)	30	5.6%	99	15.3%	24	6.9%
Class II obesity (35–39.99)	5	0.9%	9	1.4%	15	4.3%
Class III obesity (> 40)	2	0.4%	2	0.3%	11	3.2%
ED Diagnosis (*n. %*)						
No ED	462	86.0%	577	89.5%	130	37.6%
Anorexia Nervosa	22	4.1%	19	2.9%	53	15.3%
Bulimia Nervosa	20	3.7%	21	3.3%	62	17.9%
Binge Eating Disorder	16	3.0%	16	2.5%	60	17.3%
ED No Otherwise Specified	17	3.2%	12	1.9%	41	11.8%

#### Measures

##### The Italian version of the Three Factor Eating Questionnaire-Revised 18 (IT-TFEQ-R-18)

The Three Factor Eating Questionnaire-Revised 18 (TFEQ-R-18) is a questionnaire used worldwide to assess the three key dimensions of eating behavior: “Cognitive Restraint” (CR), “Uncontrolled Eating” (UE), and “Emotional Eating” (EE) [13, 28]. It consists of 18 items with a 4-point Likert-type response scale (from 1 = “definitely false” to 4 = “definitely true”) except for item#18 that is scaled on a 8-point Likert-type response scale (1 = “no restraint in eating” to 8 = “total restraint”)—which will be recoded during the scoring procedure. Items’ scores are summed into three scales: CR (6 items), UE (9 items), and EE (3 items) [6]. High scores on a scale reflect a higher level of that specific dimension. No total score should be computed. In this study, the Italian version of the TFEQ-R-18 (IT-TFEQ-R-18) provided good internal consistency in each scale: CR, omega = 0.820; UE, omega = 0.918; EE, omega = 0.845.

#### Statistical analyses

An Exploratory Graph Analysis (EGA) [31, 34–36] was performed to evaluate item clustering (*i.e.*, dimensionality) of the TFEQ-R-18—given its several advantages over exploratory factor-analytic techniques [31, 35, 37, 38]. The EGA highlights which items cluster together and their level of association revealing the most probable number of dimensions [31, 38].

Preliminary analyses were performed: examination of items’ normality, the presence of excessive correlations (e.g., *r* > 0.85) among them [34, 39] as well as their level of informativeness—an item may be deemed poorly informative if its standard deviation (SD) falls 2.5 standard deviations below the mean of all items [40–43].

To estimate the EGA model parameters, a 5000 parametric bootstrap procedure with polychoric correlations, *‘Louvain community detection algorithm’*, and the GLASSO method was used [31, 34, 36, 38].

Then, the TFEQ-R-18 items’ statistics were explored. First, item stability (IS) [34] evaluates the proportion of times the original dimension is exactly replicated across bootstrap resamples—it ranges from 0 (*“* = *perfect instability”*) to 1 (*“* = *perfect stability”*) and values higher than 0.80 (IS ≥ 0.80) suggest that the item could be considered ‘stable’ [36]. Second, network loadings (namely, standardized node strength) were computed to assess the contribution of each node to the coherence of the dimensions—and they should be interpreted according to the following benchmarks [38, 44]: small: λ_EGA_ < 0.15; moderate: λ_EGA_ < 0.25; large: λ_EGA_ < 0.35. The detailed procedure is provided in the supplementary materials (Supplementary 2).

### Results

#### Preliminary analyses

Univariate normality was observed for the all of the items of the TFEQ-R-18 (Table 2). Also, none of the 18 items of TFEQ-R-18 was poorly informative—each item of the TFEQ-R-18 provides sufficient variability as well as a good level of informativeness (i.e., *SD*_*item*_ < 2.5 SD below the mean level of informativeness, *M*_SD_ = 0.072 ± 0.705). Moreover, none of the bivariate correlations exceeded a critical level (*r* ≥ 0.82) (see Figure S1 in Supplemenatry material 2).

**Table 2:** Study 1. Descriptive statistics of items and Exploratory Graph Analysis (EGA) results

	Descriptive statistics				Hp. Dim	Item stability			EGA loadings		
	Mean	SD	Skwn	K		Stab#1	Stab#2	Stab#3	Dim#1 |λ|_EGA_	Dim#2 |λ|_EGA_	Dim#3 |λ|_EGA_
Item#1	2.16	0.850	0.229	− 0.672	UE	**1.000**	0.000	0.000	**0.288**	0.000	0.064
Item#2	1.92	0.897	0.516	− 0.811	CR	0.000	**1.000**	0.000	− 0.002	**0.343**	0.024
Item#3	2.24	1.010	0.172	− 1.145	EE	0.000	0.000	**1.000**	0.048	0.019	**0.419**
Item#4	1.83	0.899	0.796	− 0.320	UE	**0.996**	0.000	0.004	**0.263**	0.000	0.149
Item#5	1.95	0.822	0.400	− 0.696	UE	**1.000**	0.000	0.000	**0.280**	0.001	0.028
Item#6	2.07	0.939	0.372	− 0.921	EE	0.000	0.000	1.000	0.102	0.001	**0.481**
Item#7	2.16	0.878	0.134	− 0.932	UE	**1.000**	0.000	0.000	**0.295**	0.000	0.025
Item#8	1.87	0.897	0.683	− 0.518	UE	**1.000**	0.000	0.000	**0.349**	0.000	0.020
Item#9	1.91	0.956	0.647	− 0.715	EE	**1.000**	0.000	0.000	**0.308**	0.027	0.010
Item#10	1.83	0.883	0.684	− 0.586	UE	0.000	0.000	**1.000**	0.140	0.000	**0.271**
Item#11	1.97	0.907	0.372	− 1.041	CR	0.000	**1.000**	0.000	0.001	**0.514**	0.000
Item#12	1.94	0.929	0.540	− 0.811	CR	0.000	**1.000**	0.000	0.012	**0.420**	0.000
Item#13	1.85	0.827	0.594	− 0.487	UE	**1.000**	0.000	0.000	**0.341**	0.007	0.010
Item#14	2.17	0.700	0.434	0.340	UE	**0.992**	0.000	0.008	**0.180**	0.014	0.090
Item#15	2.59	1.009	− 0.150	− 1.059	CR	0.000	**1.000**	0.000	0.008	**0.165**	0.000
Item#16	2.18	0.844	0.253	− 0.593	CR	0.000	**1.000**	0.000	0.013	**0.283**	0.001
Item#17	1.72	0.836	0.798	− 0.467	UE	**0.998**	0.000	0.002	**0.228**	0.001	0.095
Item#18*	2.15	0.867	0.181	− 0.843	CR	0.000	**1.000**	0.000	0.002	**0.310**	0.000

*Recoded into a 4-point Likert-type scale according to the original validation article; Skwn.: Skewness; K: kurtosis; Hp. Dim: hypothesized dimension; Stability#(…): stability of the item (5000 replication) on the EGA-based dimension; Dim#(…): EGA-based dimension;|λ|_EGA_: absolute value of the network loading. Higher network loadings and stabilities are highlighted in bold

#### Exploratory graph analysis (EGA)

**Fig. 1:**
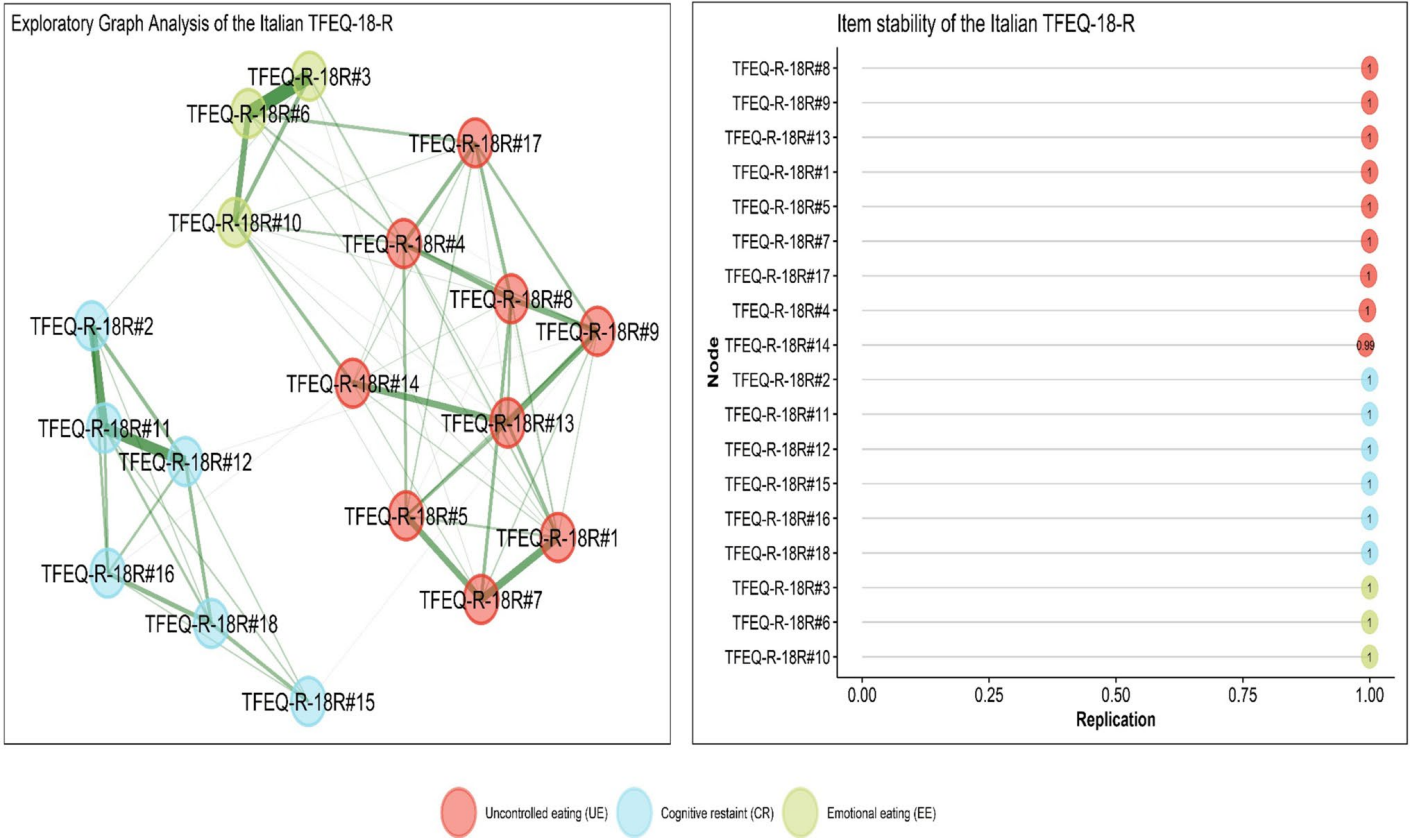
Study 1. Exploratory graph analysis (EGA) of Three Factor Eating Questionnaire-Revised-18 (TFEQ-R-18)

The bootstrapped EGA correctly identified a three-dimension/factor solution: median_bootstrapped_dimensions_ = 3; SE_bootstrapped_dimensions_ = 0.014; lower CI_bootstrapped_dimensions_ = 2.978 and upper CI_bootstrapped_dimensions_ = 3.028 and an edge density equal to 0.503 (Table 1 and Fig. 1)—with a probability of a three dimension/factor solution equal to 0.999.

#### Item statistics

The IS analysis (Table 1; Fig. 1) showed that most items were perfectly stable in the designated dimension—with a strong replication index. The first dimension (UE) displayed items with a IS higher than 0.99, items in the second dimension (CR) displayed a IS equal to 1 (perfect stability). Also, the third dimension (EE) displayed a IS equal to 1 (perfect stability). On average, items were almost perfectly stable within their designated dimension: Dim#1_IS_replication_mean_ = 0.998; Dim#2_IS_replication_mean_ = 1.00; Dim#3_IS_replication_mean_ = 1.00.

Then, EGA-based network loadings (λ_EGA_) showed a high association between items and their dimension. Considering the UE dimension, λ_EGA_ ranged from 0.180 (moderate) to 0.349 (large). For the CR dimension, λ_EGA_ ranged from 0.165 (moderate) to 0.514 (large). For the EE dimension, λ_EGA_ ranged from 0.271 (large) to 0.481 (large).

## Study 2—factorial structure of the Italian version of the TFEQ-R-18

### Methods and materials

#### Sample size calculation

In line with previous research and as suggested by the existing literature (e.g., Kline, 2023), the “*n:q* criterion” was employed to determine the minimum sample size [45]. A ratio of five individuals per parameter (5:1) was guaranteed. Thus, considering that model parameters were 75, then a minimum sample of 375 was recruited.

#### Procedure

A different sample from Study 1 was recruited but the same procedure and inclusion/exclusion criteria of Study 1 were applied. In line with previous studies [8, 15, 46, 47], participants from the general population were enrolled via social media platforms. All procedures were approved by the Ethics Committee of the IRCCS Istituto Auxologico Italiano - protocol number 2020_02_18_04.

#### Participants

The overall sample comprised 645 participants from the general population. The sample, it included 171 males (26.5%) and 474 females (73.5%), aged between 18 and 87 years (*mean* = 33.18, *SD* = 15.82), with BMIs ranging from 15.06 to 41.87 kg/m^2^ (*mean* = 23.44 kg/m^2^, *SD* = 5.421). More details are reported in Table 1.

#### Measures

Participants' demographic information, such as age and gender, as well as clinical data including weight and height (used to calculate BMI), were gathered. Additionally—together with the TFEQ-R-18—the Italian version of the following self-report questionnaires was administered:

##### The measure of eating compulsivity10 (MEC10-IT)

The MEC10 [15, 48] is a brief, valid, and accurate self-report questionnaire for the assessment of compulsive eating. Also, the MEC10-IT demonstrated high accuracy, sensibility and specificity in identifying individual with or without binge eating disorder [15]. It is comprising 10 items assessing the presence of compulsive eating patterns related to uncontrollability, urgency to eat, and binge eating behaviors. Higher scores indicate a higher degree of eating compulsivity. In this study, the internal consistency of the MEC10-IT was: omega = 0.953.

##### The Dutch Eating Behavioral questionnaire (DEBQ)

The DEBQ [49] is a 33-item self-report questionnaire designed to assess behaviors and attitudes related to eating disorders. It is widely used in both non-clinical [50, 51] and clinical samples [52]. The questionnaire comprises three dimensions: emotional eating (EE), restrained eating (RE), and external eating (ExE). In the current study, the omegas for the RE subscale, the EE subscale, and the ExE subscale were found to be 0.972, 0.949, and 0.887, respectively.

##### The eating disorder examination questionnaire (EDE-Q)

The EDEQ [53] is a 28-item self-report measure of ED psychopathology and behaviors in both community and clinical populations. The questions concern the frequency of key behavioral features of EDs in which the person engages over the preceding 28 days. The questionnaire is composed of four subscales: restraint (R); eating concern (EC); shape concern (SC); and weight concern (WC). In the present sample, the EDE-Q showed satisfactory internal consistency; R: omega = 0.838; EC: omega = 0.868; SC: omega = 0.935; WC: omega = 0.821.

#### Statistical analysis

A three correlated-factors model was specified. The Diagonally Weighted Least Squares (DWLS) estimator was used to assess the factorial structure of the TFEQ-R-18 [45, 54, 55]. Model fit was assessed by means of the classical goodness-of-fit indices (χ^2^, RMSEA, CFI, SRMR) and their recommended cutoff values: (A) statistically non-significance of the χ.^2^, (B) an RMSEA lower than 0.08, (C) a CFI higher than 0.95, and (D) an SRMR lower than 0.08 [45, 54, 56]

The internal consistency of each factor was evaluated with McDonald’s omega [50]. The item-total correlation (adjusted) and Pearson correlation coefficient (to assess convergent validity) were computed interpreted using Cohen’s benchmarks [57]: *r* < 0.10, trivial; *r* from 0.10 to 0.30, small; *r* from 0.30 to 0.50, moderate; *r* > 0.50; large. The detailed procedure is provided in the supplementary materials (Supplementary 2).

### Results

#### Structural validity

The three first-order factor model of the TFEQ-R-18 showed a good fit to the data. Despite the Chi-square statistic resulted to be statistically significant [S-Bχ^2^ (132) = 482.794; *p* < 0.001], the other fit indices revealed a good fit to the data: the RMSEA = 0.064; 90%CI 0.058–0.070; *p* (RMSEA < 0.05) < 0.001, the CFI = 0.989, the SRMR = 0.062. All the items’ loadings were statistically significant and ranged from 0.412 (item#15; CR) to 0.939 (item#6; EE)—Table 3. Also, the degree of explained variance ranged from ranged from 0.170 (item#15; CR) to 0.981 (item#6; EE). Moreover, standardized latent covariances showed that the CR scale had a small association with both the UE scale and the EE scale: *r* = 0.090 with *p* < 0.001 and *r* = 0.125 with *p* < 0.001. Conversely standardized latent covariances showed a strong—but non excessive (*r* < 0.80)—association between the UE scale and the EE scale: *r* = 0.778 with *p* < 0.001.

**Table 3:** Study 2. Item descriptive statistics, psychometric properties, and confirmatory factor analysis (CFA) results

	Descriptive statistics					IT-TOT(*r*_Adj_)			CFA	
	Mean	Median	SD	SK	K	CR	UE	EE	λ	*R*^*2*^
Item#2	1.97	2	0.968	0.553	− 0.857	0.596			0.743	0.552
Item#11	2.05	2	1.039	0.487	− 1.052	0.646			0.855	0.731
Item#12	2.27	2	1.078	0.228	− 1.243	0.657			0.810	0.656
Item#15	2.63	3	0.992	-0.184	− 1.001	0.339			0.412	0.170
Item#16	2.25	2	0.911	0.280	− 0.719	0.535			0.637	0.406
Item#18*	2.24	2	0.873	0.257	− 0.740	0.575			0.661	0.437
Item#1	2.22	2	0.881	0.251	− 0.676		0.625		0.718	0.515
Item#4	1.91	2	1.002	0.729	− 0.675		0.669		0.817	0.667
Item#5	2.00	2	0.905	0.428	− 0.832		0.620		0.721	0.519
Item#7	2.10	2	0.939	0.400	− 0.811		0.663		0.763	0.583
Item#8	1.89	2	0.986	0.772	− 0.561		0.739		0.824	0.679
Item#9	1.84	2	0.989	0.852	− 0.481		0.710		0.814	0.663
Item#13	1.87	2	0.939	0.787	− 0.392		0.712		0.808	0.653
Item#14	2.25	2	0.808	0.454	− 0.138		0.570		0.644	0.415
Item#17	1.83	2	0.908	0.724	− 0.578		0.600		0.731	0.534
Item#3	2.40	2	1.004	0.045	− 1.086			0.740	0.840	0.705
Item#6	2.18	2	1.047	0.353	− 1.108			0.801	0.939	0.881
Item#10	1.99	2	0.989	0.566	− 0.853			0.747	0.888	0.788

*Recoded into a 4-point Likert-type scale according to the original validation article; Skwn.: Skewness; K: kurtosis; all *p*-values are < 0.001. IT-TOT: item-total correlation (adjusted); λ: standardized factor loading; *R*^2^: explained variance*.* To facilitate interpretation, items are sorted according to the dimensions to which they belong

#### Psychometrics properties

Reliability analysis revealed satisfying results. Indeed, for the *CR* scale, omega was equal to 0.848, for the *UE* scale omega was equal to 0.919, and for the *EE* scale, omega was equal to 0.880.

Considering the CR scale, moderate-to-large correlations were found with the DEBQ’s RE scale (*r* = 0.825, *p* < 0.001) and EDEQ’s R scale (*r* = 0.630; *p* < 0.001). Considering the UE scale, moderate-to-large correlations were found with the MEC10 (*r* = 0.766, *p* < 0.001), the DEBQ’s EE scale (*r* = 0.649, *p* < 0.001), the DEBQ’s ExE scale (*r* = 0.594, *p* < 0.001), and EDEQ’s EC scale (*r* = 0.510; *p* < 0.001). Considering the EE scale, moderate-to-large correlations were found with the MEC10 (*r* = 0.691, *p* < 0.001), the DEBQ’s EE scale (*r* = 0.830, *p* < 0.001), EDEQ’s EC scale (*r* = 0.488; *p* < 0.001), the EDEQ’s SC scale (*r* = 0.487, *p* < 0.001), and the EDEQ’s WC scale (*r* = 0.441, *p* < 0.001). Results are reported in Table 4.

**Table 4:** Study 2. Correlation among variables

		Descriptive		Correlations									
		M	SD	1	2	3	4	5	6	7	8	9	10
1	Cognitive Restraint	13.42	4.153	-									
2	Uncontrolled Eating	17.92	6.178	.074	-								
3	Emotional Eating	6.57	2.723	.099^*^	.676^***^	-							
4	Eating Compulsivity	11.25	9.430	.285^***^	.766^***^	.691^***^	-						
5	Restrained Eating	2.72	0.991	.825^***^	.222^***^	.227^***^	.360^***^	-					
6	Emotional Eating	2.31	0.998	.243^***^	.649^***^	.830^***^	.711^***^	.324^***^	-				
7	External Eating	2.96	0.688	.074	.594^***^	.387^***^	.422^***^	.138^***^	.443^***^	-			
8	Eating Restraint	1.43	1.376	.630^***^	.279^***^	.211^***^	-.165	.747^***^	.360^***^	.084	-		
9	Eating Concern	0.86	1.167	.310^***^	.510^***^	.488^***^	.555^**^	.498^***^	.483^***^	.255^***^	.529^***^	-	
10	Shape Concern	2.35	1.641	.369^***^	.409^***^	.487^***^	.299	.577^***^	.488^***^	.232^***^	.610^***^	.738^***^	-
11	Weight Concern	1.95	1.480	.409^***^	.406^***^	.441^***^	.510^***^	.585^***^	.495^***^	.253^***^	.616^***^	.751^***^	.907^***^

*p < 0.020; **p < 0.010; ***p < 0.001

## Study 3—assessing TFEQ-R-18 mean differences across EDs conditions

### Methods and materials

#### Sample size calculation

The minimum sample size was computed a priori by using the G*Power software [58]. The multivariate analysis of variance (MANOVA) family of statistics was chosen—specifying five groups of ED condition (No ED vs. AN vs. BN vs. BED vs. Other disordered eating condition) and the 3 scales of the TFEQ-R-18 as response variables.. The a priori minimum desired statistic (Pillai’s trace; V) was set to 0.2 (small effects)—resulting in: *f*^2^(V) = 0.071 [59, 60]—the Type I error (α) was set at 0.05 (two-sided), and the Power (1 – β) was set at 0.95. Consequently, an overall sample of 125 subjects was required – 25 participants per group.

#### Procedure

A different sample from Study 1 and Study 2 was recruited but the same procedure and inclusion/exclusion criteria of Study 1 and Study 2 were applied. Also in this case, social media platforms (e.g., Facebook, Twitter/X, etc.) were used to enroll participants form the general population. All procedures were approved by the Ethics Committee of the IRCCS Istituto Auxologico Italiano - protocol number 2020_02_18_04.

#### Participants

The overall sample comprised 346 participants. The sample included 56 males (16.2%) and 290 females (83.8%), aged between 18 and 83 years (*mean* = 36.23, *SD* = 14.51), with BMIs ranging from 13.72 to 58.83 kg/m^2^ (*mean* = 24.88 kg/m^2^, *SD* = 6.32). More in detail, 130 (37.6%) participants reported to have any EDs (No EDs), 53 (15.3%) reported a diagnosis of Anorexia Nervosa (AN), 62 (17.9%) a diagnosis of Bulimia Nervosa (BN); 60 (17.3%) a diagnosis of Binge Eating Disorder (BED), and 41 (11.8%) Other disordered eating conditions. More details are reported in Table 1.

#### Measures

A demographic information form assessing participants' demographic information (e.g., age, gender, diagnosis of EDs, weight and height) and the Italian TFEQ-R-18 were administered.

#### Statistical analysis

First, preliminary analysis were performed, normality, linearity, multicollinearity, and homogeneity of covariance matrices [39]. Then a Multivariate Analysis of Variance (MANOVA) was performed to determine possible differences between ED conditions (No ED vs. AN vs. BED vs. Other disordered eating condition—independent variable) simultaneously on the TFEQ-R-18 scales (dependent variables). The advantage of employing MANOVA over conducting three separate analyses of variance lies in the multivariate statistical method's capability to mitigate Type I error inflation (i.e., rejecting the null hypothesis when it is true). Wilks’ lambda (Λ) was chosen to test the multivariate effect. Moreover, focused contrasts with Bonferroni’s correction were performed. Partial eta-square (η^2^_p_) and Cohen’s *d* were used to quantify the difference in multiple and pairwise comparisons, respectively—with following benchmarks: small (η^2^_p_: 0.011 to 0.059; *d*: 0.20 to 0.49), moderate (η^2^_p_: 0.060 to 0.139; *d*: 0.50 to 0.79), and large (η^2^_p_ > 0.140; *d* > 0.80) [57].

### Results

#### Preliminary analysis

The raw score of each variable was almost normally distributed and their relationships were substantially linear. Tolerance and variance inflation factor (VIF) statistics revealed the absence of multicollinearity (Table S1 — Supplementary materials 2). The Box’s *M* resulted to be statistically significant (*M* = 66.809, *F* = 2.672, *p* < 0.001)—however, it should be noted that MANOVA is robust to small violations of assumptions [39, 61]. Thus, considering these results, the MANOVA was performed.

#### Multivariate analysis of variance

**Fig. 2:**
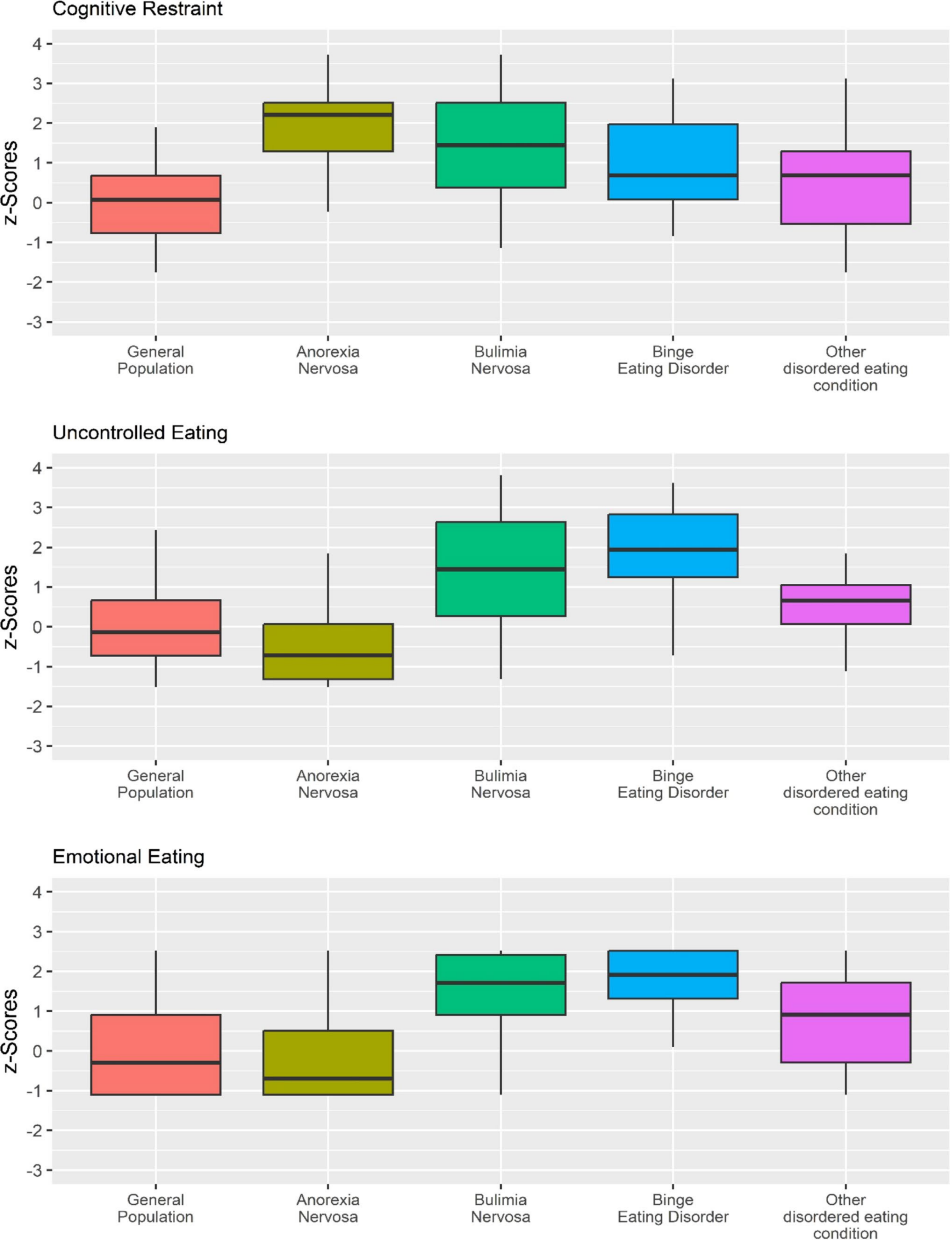
Study 3. Boxplot. For better interpretation, the graph displays standardized values (*z*-scores) centered on subjects without any eating disorder (No ED)

A statistically significant multivariate effect was found: Λ = 0.404, *F* = 30.351, *p* < 0.001; η^2^_p_ = 0.261 (large effect size) with a statistically significant between groups difference for the CR scale [*F* = 35.261, *p* < 0.001, η^2^_p_ = 0.293 (large effect size)], as well as the UE scale [*F* = 52.183, *p* < 0.001, η^2^_p_ = 0.380 (large effect size)], and the EE scale [*F* = 48.693, *p* < 0.001, η^2^_p_ = 0.364 (large effect size)]—Fig. 2. Detailed results are reported in Table 5.

**Table 5:** Study 3. Focused t-test results

Target group		Comparison group		Statistics		
Cognitive restraint						
	M (SD)		M (SD)	t	*p*-value	*d*
No ED	11.75 (3.285)	AN	18.25 (3.408)	− 10.565	< .001	− 1.722
		BN	16.39 (4.546)	− 7.962	< .001	− 1.229
		BED	14.68 (3.753)	− 4.978	< .001	− 0.777
		ED other	13.10 (4.358)	− 1.990	0.474	− 0.356
AN	18.25 (3.408)	BN	16.39 (4.546)	2.635	0.088	0.493
		BED	14.68 (3.753)	5.012	< .001	0.945
		ED other	13.10 (4.358)	6.565	< .001	1.365
BN	16.39 (4.546)	BED	14.68 (3.753)	2.495	0.131	0.452
		ED other	13.10 (4.358)	4.334	< .001	0.872
BED	14.68 (3.753)	ED other	13.10 (4.358)	2.076	0.387	0.421

Uncontrolled eating						
	M (SD)		M (SD)	t	*p*-value	*d*
No ED	16.66 (5.068)	AN	15.00 (6.013)	1.834	0.675	0.299
		BN	24.00 (7.165)	− 8.552	< .001	− 1.320
		BED	26.70 (5.036)	− 11.569	< .001	− 1.806
		ED other	19.83 (4.236)	− 3.181	0.016	− 0.570
AN	15.00 (6.013)	BN	24.00 (7.165)	− 8.654	< .001	− 1.619
		BED	26.70 (5.036)	− 11.164	< .001	− 2.105
		ED other	19.83 (4.236)	− 4.177	< .001	− 0.869
BN	24.00 (7.165)	BED	26.70 (5.036)	− 2.682	0.077	− 0.486
		ED other	19.83 (4.236)	3.727	0.002	0.750
BED	26.70 (5.036)	ED other	19.83 (4.236)	6.099	< .001	1.236

Emotional eating						
	M (SD)		M (SD)	t	*p*-value	*d*
No ED	5.74 (2.486)	AN	5.25 (2.464)	1.211	1.000	0.197
		BN	9.18 (2.671)	− 8.915	< .001	− 1.376
		BED	10.05 (2.086)	− 11.052	< .001	− 1.725
		ED other	7.63 (2.853)	− 4.234	< .001	− 0.758
AN	5.25 (2.464)	BN	9.18 (2.671)	− 8.409	< .001	− 1.573
		BED	10.05 (2.086)	− 10.198	< .001	− 1.922
		ED other	7.63 (2.853)	− 4.595	< .001	− 0.956
BN	9.18 (2.671)	BED	10.05 (2.086)	− 1.928	0.547	− 0.349
		ED other	7.63 (2.853)	3.067	0.023	0.617
BED	10.05 (2.086)	ED other	7.63 (2.853)	4.770	< .001	0.967

All *p*-values are Bonferroni corrected. No ED: any eating disorder (*n* = 130); AN: Anorexia Nervosa (*n* = 53); BN: Bulimia Nervosa (*n* = 62); BED: Binge Eating Disorder (*n* = 60); ED other: Other disordered eating condition (*n* = 41); *t*:* t*-test; *d*:  Cohen’s *d* (effect size.)

## General discussion

Feeding and Eating Disorders along with disordered eating behaviors and obesity, represent serious issues resulting that significantly impact on the individual's physical and psychological health as well as social functioning. The lifetime prevalence that is constantly increasing [62–66] along with related psychological conditions such as anxiety and depression [67] and medical conditions such as obesity [68, 69]. Therefore, a thorough and comprehensive understanding of the cognitive and emotional aspects of eating behaviors is essential for the development of effective interventions [2].

One of the most widely used measures in the field of eating behavior research is the TFEQ-R-18 [6]. It serves as a robust and concise assessment tool for measuring the psychological aspects of eating behavior, making it valuable for both clinical and research purposes. Since the TFEQ-R-18 is not currently available in Italian, this three-step study aimed to validate it by testing its item clustering, factorial structure, investigating its psychometric properties, and assessing its performance in three distinct large community samples.

In Study 1, the Exploratory Graph Analysis (EGA), utilizing a data-driven approach, clearly confirmed that the items of the TFEQ-R-18 clusters in three distinct dimensions (with a probability of 0.999): cognitive restraint, uncontrolled eating, and emotional eating. Specifically, each item loaded onto the correct dimension with robust indexes of item stability, replication, and EGA loadings. Results from the EGA also provided evidence for the construct validity of the TFEQ-R-18 dimensions [38]. Despite correlations among dimensions, each item grouped with its theoretically hypothesized dimension. All dimensions remained distinctly independent and separate within the overall structure, reflecting an ideal outcome. Furthermore, EGA findings illuminated the interrelationships between the items and the three dimensions [34, 37]. A precise graphical representation showcased these connections for interpretation. All dimensions of the questionnaire exhibit mutual associations, with stronger connections between the two dimensions related to food intake (UE-EE: *r* = 0.623) and weaker connections with the CR dimension (CR-UE: *r* = 0.121; CR-EE: *r* = 0.133). Subsequently, the stability of the items was assessed [36]. Examining the stability of items and dimensions provides valuable insights into dimension instability, such as misallocation, and multidimensionality. The analysis demonstrated that the items in the TFEQ-R-18 displayed stability within their intended dimensions and across them.

In Study 2, the structural validity of the TFEQ-R-18 was further confirmed through Confirmatory Factor Analysis (CFA), revealing a three first-order factor structure that exhibited good fit indices to the data. All items displayed robust loadings on the hypothesized latent factors, indicating their effective representation of the underlying constructs [45, 54]. Furthermore, concerning its psychometric characteristics, the TFEQ-R-18 demonstrated high internal consistency. The convergent validity analyses showed meaningful correlations. First, the CR scale showed significant associations with both the DEBQ Restrained scale (*r* = 0.825) and the EDEQ Restraint eating scale (*r* = 0.630). These findings provide robust evidence of associations between cognitive efforts to regulate food intake to control weight and body shape (CR) and behavioral dimensions related to reduced food intake [70–73]. Second, the UE scale demonstrated strong associations with both the MEC10 (*r* = 0.766) and the DEBQ External Eating scale (*r* = 0.594). This suggests that the tendency to lose control and overeat (UE) is strongly associated with addiction-like eating behaviors and overeating [8, 15]. Lastly, the EE scale showed strong associations with both the MEC10 (*r* = 0.691) and the DEBQ Emotional Eating scale (*r* = 0.830). This suggests that the tendency to eat in response to emotional urges (EE) is strongly associated with binge eating and addiction-like eating behaviors [8, 15]. It should be noted that UE and EE were strongly correlated (*r* = 0.676), suggesting that the use of food as a coping strategy in response to dysregulated emotional stimuli (EE) may be a strong predictor of binge-related behaviors (UE) and overeating [2, 8, 15, 74–76].

Study 3 showed that individuals with different eating disorder conditions exhibit varying levels of response in the three scales of the TFEQ-R-18. Participants with no EDs displayed low levels of CR, UE, and EE. It's worth noting, however, that for all three scales, considerable variability was observed. This suggests that none of these constructs are necessarily related exclusively to pathological aspects but rather measure aspects that are also present in the general population. If chronic and/or highly persistent, these aspects may serve as indicators of eating disorders [73, 75–77]. Considering individuals with AN, they report the highest levels of CR and, simultaneously, low levels of UE and EE. These findings align with Study 2, where concerns about body weight and shape and control over food would be manifested through cognitive rigidity and narrowness [78–80]. Considering individuals with BN and BED, despite some slight differences, they exhibit elevated scores in CR, accompanied by high scores in UE and EE. These findings align with existing literature and suggest that individuals with BN or BED would combine or alternate states of high cognitive rigidity and control with an inability to regulate emotional state [2, 74, 81, 82]. This inability manifests in dysfunctional eating behaviors and the use of food as an external regulator [2, 51, 52, 83–87].

### Clinical implications and future perspectives

The TFEQ-R-18 is a valuable assessment tool for clinicians because of its ability to measure transdiagnostic constructs shared by various psychological disorders and difficulties of different levels of severity [77, 81, 88, 89]. Assessing uncontrolled eating, emotional eating, and cognitive restraint dimensions is crucial, as they can play multiple roles and act as causes, results, or maintenance factors of dysfunctional eating patterns [2, 88]. This is particularly relevant in the realm of eating disorders, providing crucial information for both the conceptualization and treatment of clinical conditions.

Regarding the implications of research in the clinical context, exploring the characteristics and associations of the TFEQ-R-18 across various psychopathological profiles (e.g., traumatized individuals, those with health-related issues, personality disorders, or eating disorders) would be intriguing. Future studies, employing cross-cultural and longitudinal designs, will provide independent replication and assessment of the psychometric properties and factorial structure of the TFEQ-R-18 in different samples (clinical vs. non-clinical) and age groups (e.g., adolescents).

The present research makes an incremental contribution by offering the first validation of the TFEQ-R-18 in the Italian language, thus making it accessible for researchers and clinicians involved in preventive actions or working with individuals exhibiting dysfunctional eating behavior.

## Strength and limits

Certain limitations of this study can provide valuable insights for future research. A primary limitation is the exclusive use of self-report measures and the cross-sectional design, which hinders the ability to assess changes in the tool over time or its predictive validity (e.g., test–retest reliability and longitudinal measurement invariance). Moreover, regarding Study 2, despite the use of robust and reliable estimation methods (i.e., DWLS [90]) and a sample of moderate/large size (*N* = 645—approximately 9 participants per parameter), it is still possible that this sample size may be insufficient to yield fully reliable results. Arguably, employing a participant-to-parameter ratio of 10:1 might have led to more robust outcomes. Future studies could consider attempting to replicate these findings with even larger sample sizes. Furthermore, future studies should assess measurement invariance across different groups of disordered eating conditions. Additionally, future studies could delve into identifying recurrent patterns of cognitive restraint, uncontrolled eating, and emotional eating by establishing latent psychological profiles.

Despite the aforementioned limitations, this contribution has notable methodological and clinical strengths. It marks the first attempt to explore the psychometric properties of the TFEQ-R-18 in the Italian general population, and the results successfully confirmed the reliability and validity of the tool, relying on robust and internationally recommended statistical methods. In terms of methodological strengths, this research employed a combination of EGA and CFA to examine the latent dimensional structure of the scale [38]. EGA is an innovative method that offers the advantage of accurately assessing the underlying dimensional structure of a tool with high precision, surpassing other methods that require the pre-specification of the number of dimensions [37]. Moreover, EGA provides a visual network plot, showcasing item clustering, their levels of association, and the number of dimensions to retain, leading to valuable psychometric and clinical interpretations [31]. Subsequently, the CFA confirmed the results obtained from the EGA. Therefore, the TFEQ-R-18 can be effectively employed for both clinical and research purposes, accurately measuring popular, important, and transdiagnostic dimensions of eating disorders—making it a viable alternative to lengthier questionnaires.

The TFEQ-R-18 proves to be a dependable tool for assessing the presence and intensity of uncontrolled eating, emotional eating, and cognitive restraint in three large community samples. The questionnaire exhibited good construct validity and reliability, measuring transdiagnostic characteristics of various eating-related conditions. It is important to emphasize that none of these constructs (cognitive restraint, uncontrolled eating, and emotional eating) are (is) necessarily associated with psycho-pathological aspects; rather, they are present in the general population with varying levels of variability, prevalence, and intensity [2]. Therefore, this questionnaire aims to measure variables present in the general population that would help deepen the clinical/diagnostic examination, assisting the clinician in formulating diagnostic hypotheses. As a result, the TFEQ-R-18 can be readily utilized by clinicians and researchers to support the development of targeted interventions, promoting better psychological health and addressing dysfunctional eating patterns.

## What is already known on this subject?

- Eating disorders and dysfunctional eating behaviors are increasingly prevalent in the population.
- The revised Three-Factor Eating Questionnaire 18 (TFEQ-R-18) is a widely used tool worldwide.
- The TFEQ-R-18 has demonstrated good psychometric properties, and its brevity allows for its use in both clinical and research settings.

## What does this study add?

- The present study aimed to validate the Italian version of the TFEQ-R-18 in three large community samples.
- This study aimed to test—for the first time—psychometric properties of the TFEQ-R-18 using both novel techniques (e.g., exploratory graph analysis) and well established statistical analysis such as confirmatory factor analysis.
- The factorial structure of the TFEQ-R-18 was successfully replicated across two independent studies (Study 1 and Study 2) and in Study 3 differences among eating disorder diagnostic clusters were analyzed.

### Supplementary Information

Below is the link to the electronic supplementary material.

Supplementary file1 (PDF 119 KB)

Supplementary file2 (PDF 526 KB)

## Data Availability

The datasets generated during and/or analyzed during the current study are available from the corresponding author on reasonable request.
