# Supplementary material for: Cognitive restraint, uncontrolled eating, and emotional eating. The Italian version of the Three Factor Eating Questionnaire-Revised 18 (TFEQ-R-18): a three-step validation study
Source: Eat Weight Disord. 2024 Feb 24;29(1):16. doi: 10.1007/s40519-024-01642-y (PMC10894126; doi:10.1007/s40519-024-01642-y)
Supplement: Supplementary file 1 — Supplementary file1 (PDF 119 KB) [file 40519_2024_1642_MOESM1_ESM.pdf]

Three Factor Eating Questionnaire-R-18

ISTRUZIONI
Troverà qui di seguito una lista di affermazioni. La preghiamo di **rispondere a tutte le domande** ponendo un segno in corrispondenza del valore numerico – compreso **tra 1 (COMPLETAMENTE FALSO) e 4 (COMPLETAMENTE VERO)** – che meglio indica la frequenza con la quale prova o fa esperienza delle affermazioni sottostanti.

PARTE I

1	2	3	4
**Completamente FALSO**	**Prevalentemente FALSO**	**Prevalentemente VERO**	**Completamente VERO**

**1**	Quando sento il profumo di un cibo delizioso, mi è difficile trattenermi dal mangiarlo, anche se ho appena finito di mangiare.	**1**	**2**	**3**	**4**
**2**	Mangio delle porzioni piccole appositamente, come sistema per controllare il mio peso.	**1**	**2**	**3**	**4**
**3**	Quando sono ansioso/a mi ritrovo a mangiare.	**1**	**2**	**3**	**4**
**4**	A volte, quando inizio a mangiare, mi sembra impossibile fermarmi.	**1**	**2**	**3**	**4**
**5**	Quando sono in compagnia di qualcuno che sta mangiando, spesso mi sento così affamato che mangio anche io.	**1**	**2**	**3**	**4**
**6**	Quando sono giù di morale, spesso mangio in modo eccessivo.	**1**	**2**	**3**	**4**
**7**	Quando vedo una vera squisitezza, spesso mi viene così fame che devo mangiare subito.	**1**	**2**	**3**	**4**
**8**	Mi viene così tanta fame che spesso il mio stomaco sembra un pozzo senza fondo.	**1**	**2**	**3**	**4**
**9**	Ho sempre fame, per cui è difficile per me smettere di mangiare prima di avere finito il cibo nel piatto.	**1**	**2**	**3**	**4**
**10**	Quando mi sento solo/a, mi consolo mangiando.	**1**	**2**	**3**	**4**
**11**	Mi trattengo di proposito ai pasti, in modo da non aumentare di peso.	**1**	**2**	**3**	**4**
**12**	Non mangio certi cibi perché mi fanno ingrassare.	**1**	**2**	**3**	**4**
**13**	Ho sempre abbastanza fame da mangiare in qualsiasi momento.	**1**	**2**	**3**	**4**

Rossi, AA et al. (2024). Cognitive Restraint, Uncontrolled Eating, and Emotional Eating. The Italian Version of the Three Factor Eating Questionnaire – Revised 18 (TFEQ-R-18): A Three-step Validation Study. *Eating and Weight Disorders*.

PARTE II

**14**	Quanto spesso hai fame?<br>**1.** Solo ai pasti;<br>**2.** Qualche volta tra un pasto e l’altro;<br>**3.** Spesso tra un pasto e l’altro;<br>**4.** Quasi sempre;

**15**	Quanto spesso eviti di “fare scorte” di cibi allettanti?<br>**1.** Quasi mai;<br>**2.** Raramente;<br>**3.** Abbastanza;<br>**4.** Quasi sempre;

**16**	Quanto è probabile che tu mangi – consapevolmente – meno di quanto vorresti?<br>**1.** Improbabile;<br>**2.** Poco probabile;<br>**3.** Abbastanza probabile;<br>**4.** Molto probabile;

**17**	Ti capita di pensare di abbuffarti, anche se non hai fame?<br>**1.** Mai;<br>**2.** Raramente;<br>**3.** Qualche volta;<br>**4.** Una volta a settimana;

**18**	Su una scala da 1 a 8, dove 1 significa “nessuna restrizione nel mangiare” (mangiare ciò che vuoi quando vuoi) e 8 significa “restrizione totale” (limitazione costante del consumo di cibo senza mai “mollare”), quale numero ti daresti?<br>**1 – 2 – 3 – 4 – 5 – 6 – 7 – 8**<br>(nessuna restrizione) (restrizione totale)
