# Supplementary material for: Cognitive restraint, uncontrolled eating, and emotional eating. The Italian version of the Three Factor Eating Questionnaire-Revised 18 (TFEQ-R-18): a three-step validation study
Source: Eat Weight Disord. 2024 Feb 24;29(1):16. doi: 10.1007/s40519-024-01642-y (PMC10894126; doi:10.1007/s40519-024-01642-y)
Supplement: Supplementary file 2 — Supplementary file2 (PDF 526 KB) [file 40519_2024_1642_MOESM2_ESM.pdf]

Cognitive Restraint, Uncontrolled Eating, and Emotional Eating. The Italian Version of the Three Factor Eating Questionnaire – Revised 18 (TFEQ-R-18): A Three-step Validation Study

Alessandro Alberto Rossi*, Giada Pietrabissa; Gianluca Castelnuovo; Stefania Mannarini

SUPPLEMENTARY MATERIAL

STATISTICAL ANALYSIS

CORRESPONDING AUTHOR:

Alessandro Alberto Rossi
Department of Philosophy, Sociology, Education, and Applied Psychology, University of Padova, via Venezia, 12 – 35131 – Padova, Italy
Email: a.rossi@unipd.it

Index

Study 1. The dimensional structure of the Italian TFEQ-R-18 ... 3

R packages used ... 3

Statistical analyses ... 3

Figure S1. Item correlation matrix ... 5

Study 2. factorial structure and measurement invariance of the Italian version of the TFEQ-R-18 ... 6

R packages used ... 6

Statistical analysis ... 6

Study 3 – Assessing TFEQ-R-18 mean differences across EDs conditions ... 7

R packages used ... 7

Statistical analysis – preliminary analysis ... 7

References ... 8

Study 1. The dimensional structure of the Italian TFEQ-R-18

R packages used

The R software was used with the following packages: bootnet (v.1.4.3) [1]; corrplot (v. 0.84.) [2]; EGAnet (v.0.9.8) [3, 4]; igraph (v.1.2. 5) [5], networkTools (v.1.2. 3) [6], qgraph (v.1.6.5) [7] psych (v.2.0.7) [8], and psychTools (v.2.0.6) [9]

Statistical analyses

Preliminary analyses were performed before carrying out the EGA [10]. First, the normality of items, as well as the presence of excessive correlations (r > 0.70) between items, were inspected [11, 12]. Second, for each item, the level of informativeness was evaluated [13, 14]. An item should be considered as badly informative if its SD is 2.5SDs below the average of all the items [14–16]. Considering that the purpose of this study was not to reduce the number of items in the TFEQ-R-18, item redundancy was not performed [4] [10]. Consequently, an EGA [10, 17] was performed to assess item clustering (*i.e.*, dimensionality) of the TFEQ-R-18 – given the several advantages over traditional exploratory factor-analytic techniques [17–21].

The EGA produces a plot that should be considered as a 'visual guide' [17, 19, 22] displaying the correct number of dimensions – by highlighting which items cluster together and their level of association in which the thicker an edge, the strongest the relationship between an item of a specific cluster (dimension/factor) [21, 23]. The EGA was carried out by using a 5,000 parametric bootstrap procedure. Moreover, the GLASSO method with polychoric correlations was used to estimate model parameters[17, 24, 25]. In addition, the correct number of dimensions was detected by using the *'Louvain community detection algorithm'* [26], which has demonstrated better performances than the Walktrap algorithm [27] in recognizing clusters of items/dimensions [10].

Once the EGA confirmed the number of dimensions composing the TFEQ-R-18, the questionnaire and item statistics were explored. First, item stability (IS) was computed. IS evaluates the proportion of times the original dimension is exactly replicated across bootstrap resamples – thus, it assesses the occurrence of each item within a certain specific dimension [10]. IS ranging from 0 (*"= perfect instability"*) to 1 (*"= perfect stability"*) and values higher than 0.80 (IS $\geq$ 0.80) suggest that the item could be considered 'stable' and consistently identified in the dimension [28]. Subsequently, the contribution of each node to the coherence of the dimensions was then assessed using standardized node strength – namely, network loadings. It is important to note that they represent partial correlation

loadings and thus the magnitude of these loadings should be interpreted according to the following benchmarks [21, 29]: small: $\lambda_{EGA} < 0.15$; moderate: $\lambda_{EGA} < 0.25$; large: $\lambda_{EGA} < 0.35$.

Finally, correlations between items were assessed using Pearson's correlation coefficient and they were interpreted using Cohen's [30] classical benchmarks: $r < .10$, trivial; r from 0.10 to 0.30, small; r from 0.30 to 0.50, moderate; $r >$ 0.50

Figure S1. Item correlation matrix

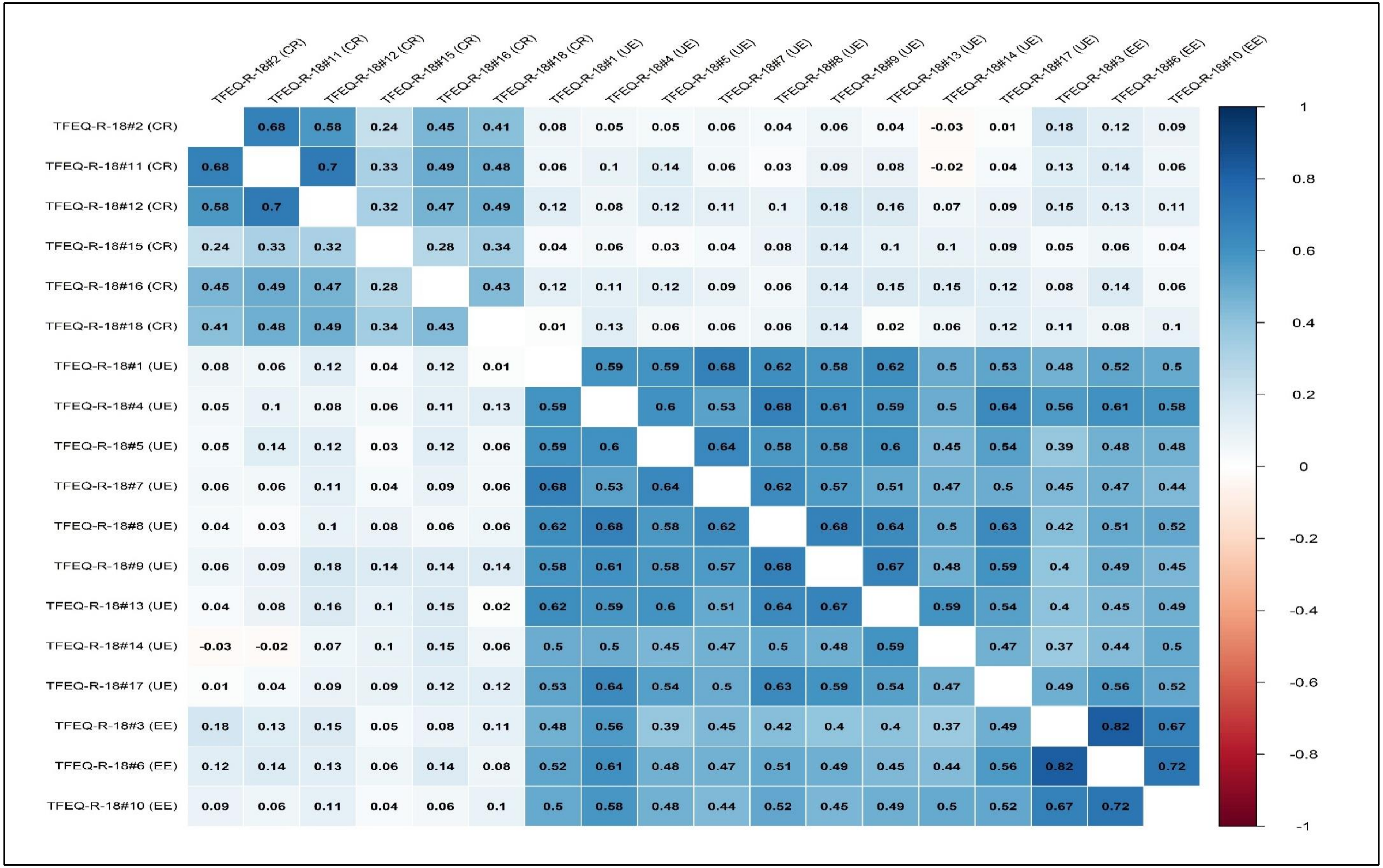

Study 2. factorial structure and measurement invariance of the Italian version of the TFEQ-R-18

R packages used

R software [31] was used to perform statistical analyses with the following packages: corrplot (v. 0.84) [2]; lavaan [32], psych (v.2.0.7) [8], and psychTools (v.2.0.6) [9].

Statistical analysis

A three correlated-factors model was specified and tested for individuals from the general population and inpatients with severe obesity, separately The diagonal weighted least square (DWLS) estimator was used to assess the factorial structure of the TFEQ-R-18 [33–36]. Model fit was assessed by means of the Satorra-Bentler Chi-square statistics (S-Bχ^2), the Root-Mean Square Error of Approximation (RMSEA), the Comparative Fit Index (CFI), and the Standardized Root Mean Residual (SRMR) [33–37]. Moreover, the following cut-off criteria were chosen to evaluate the goodness of fit: (A) statistically non-significance of the χ^2, (B) an RMSEA lower than 0.08, (C) a CFI higher than 0.95, and (D) an SRMR lower than 0.08 [33–37].

The item-total correlation (adjusted) was computed [11, 12, 38]. The internal consistency of each factor was evaluated with McDonald's Omega [39]. Convergent validity was assessed with the Pearson correlation coefficient [12] and interpreted using the aforementioned Cohen's benchmarks [30].

Study 3 – Assessing TFEQ-R-18 mean differences across EDs conditions

R packages used

R software [31] was used to perform statistical analyses with the following packages: 'esvis' [40], 'ggplot2 [41], psych (v.2.0.7) [8], psychTools (v.2.0.6) [9]. and 'tidyverse' [42].

Statistical analysis – preliminary analysis

Table 1. Descriptive statistics of questionnaires (row scores) and correlations between variables

		Descriptive				Correlations			Collinearity	
		M	SD	SK	K	1	2	3	VIF	T
1	Cognitive Restraint	14.25	4.457	0.164	-0.586	-			1.056	.947
2	Uncontrolled Eating	19.84	7.018	0.326	-0.921	.141*	-		2.198	.455
3	Emotional Eating	7.25	3.115	0.038	-1.327	.226**	.738**	-	2.271	.440

Note: * = p < 0.010; ** = p < 0.001; M = mean; SD = standard deviation; Sk = skewness; K = kurtosis; VIF = variance inflation factor; T = Tolerance.

References

1. Epskamp S, Borsboom D, Fried EI (2018) Estimating psychological networks and their accuracy: A tutorial paper. Behav Res Methods 50:195–212. https://doi.org/10.3758/s13428-017-0862-1

2. Wei, T., Simko, V. (2017) R package "corrplot": Visualization of a Correlation Matrix

3. Golino H, Christensen AP (2023) EGAnet: Exploratory Graph Analysis – A framework for estimating the number of dimensions in multivariate data using network psychometrics.

4. Christensen AP, Garrido LE, Golino H (2023) Unique Variable Analysis: A Network Psychometrics Method to Detect Local Dependence. Multivar Behav Res 0:1–18. https://doi.org/10.1080/00273171.2023.2194606

5. Csardi G, Nepusz T (2006) The igraph software package for complex network research. InterJournal Complex Systems

6. Jones P networktools: Tools for Identifying Important Nodes in Networks

7. Epskamp S, Cramer AOJ, Waldorp LJ, et al (2012) qgraph: Network Visualizations of Relationships in Psychometric Data. J Stat Softw 48:1–18. https://doi.org/10.18637/jss.v048.i04

8. Revelle W (2018) psych: Procedures for Personality and Psychological Research

9. Revelle W (2023) psychTools: Tools to Accompany the "psych" Package for Psychological Research

10. Christensen AP, Golino H, Silvia PJ (2020) A Psychometric Network Perspective on the Validity and Validation of Personality Trait Questionnaires. Eur J Personal 34:1095–1108. https://doi.org/10.1002/per.2265

11. Howell DC (2013) Statistical Methods for Psychology. Wadsworth, Cengage Learning., Belmont, CA, US

12. Tabachnick BG, Fidell LS (2014) Using Multivariate Statistics. Pearson, Harlow

13. Bottesi G, Marchetti I, Sica C, Ghisi M (2020) What is the internal structure of intolerance of uncertainty? A network analysis approach. J Anxiety Disord 75:102293. https://doi.org/10.1016/j.janxdis.2020.102293

14. Mullarkey MC, Marchetti I, Bluth K, et al (2021) Symptom centrality and infrequency of endorsement identify adolescent depression symptoms more strongly associated with life satisfaction. J Affect Disord 289:90–97. https://doi.org/10.1016/j.jad.2021.02.064

15. Marchetti I (2019) Hopelessness: A Network Analysis. Cogn Ther Res 43:611–619. https://doi.org/10.1007/s10608-018-9981-y

16. Mullarkey MC, Marchetti I, Beevers CG (2019) Using Network Analysis to Identify Central Symptoms of Adolescent Depression. J Clin Child Adolesc Psychol 48:656–668. https://doi.org/10.1080/15374416.2018.1437735

17. Golino HF, Epskamp S (2017) Exploratory graph analysis: A new approach for estimating the number of dimensions in psychological research. PLoS ONE 12:. https://doi.org/10.1371/journal.pone.0174035

18. Golino HF, Demetriou A (2017) Estimating the dimensionality of intelligence like data using Exploratory Graph Analysis. Intelligence 62:54–70. https://doi.org/10.1016/j.intell.2017.02.007

19. Golino H, Moulder R, Shi D, et al (2020) Entropy Fit Indices: New Fit Measures for Assessing the Structure and Dimensionality of Multiple Latent Variables. Multivar Behav Res 1–29. https://doi.org/10.1080/00273171.2020.1779642

20. Golino H, Shi D, Christensen AP, et al (2020) Investigating the performance of exploratory graph analysis and traditional techniques to identify the number of latent factors: A simulation and tutorial. Psychol Methods 25:292–320. https://doi.org/10.1037/met0000255

21. Christensen AP, Golino H (2021) On the equivalency of factor and network loadings. Behav Res Methods 53:1563–1580. https://doi.org/10.3758/s13428-020-01500-6

22. Panzeri A, Rossi Ferrario S, Cerutti P (2021) Psychological Differences Among Healthcare Workers of a Rehabilitation Institute During the COVID-19 Pandemic: A Two-Step Study. Front Psychol 12:

23. Mair P (2018) Modern Psychometrics with R. Springer International Publishing, Cham

24. Epskamp S (2017) Network Psychometrics. Doctoral dissertation, University of Amsterdam, NL

25. Costantini G, Epskamp S, Borsboom D, et al (2015) State of the aRt personality research: A tutorial on network analysis of personality data in R. J Res Personal 54:13–29. https://doi.org/10.1016/j.jrp.2014.07.003

26. Blondel VD, Guillaume J-L, Lambiotte R, Lefebvre E (2008) Fast unfolding of communities in large networks. J Stat Mech Theory Exp 2008:P10008. https://doi.org/10.1088/1742-5468/2008/10/P10008

27. Pons P, Latapy M (2006) Computing Communities in Large Networks Using Random Walks. J Graph Algorithms Appl 10:191–218. https://doi.org/10.7155/jgaa.00124

28. Christensen AP, Golino H (2021) Estimating the Stability of Psychological Dimensions via Bootstrap Exploratory Graph Analysis: A Monte Carlo Simulation and Tutorial. Psych 3:479–500. https://doi.org/10.3390/psych3030032

29. Golino H, Thiyagarajan JA, Sadana R, et al (2020) Investigating the broad domains of intrinsic capacity, functional ability and environment: An exploratory graph analysis approach for improving analytical methodologies for measuring healthy aging. PsyArXiv Prepr. https://doi.org/10.31234/osf.io/hj5mc

30. Cohen J (1988) Statistical Power Analysis for the Behavioral Sciences. Lawrence Erlbaum Associates., Hillsdale, NJ

31. The R-core Team (2017) R: The R Project for Statistical Computing

32. Rosseel Y (2012) lavaan: An R Package for Structural Equation Modeling. J Stat Softw 48:1–36. https://doi.org/10.18637/jss.v048.i02

33. Brown TA (2015) Confirmatory factor analysis for applied research, 2nd ed. The Guilford Press, New York, NY, US

34. Hoyle R H (2023) Handbook of Structural Equation Modeling: Second Edition. The Guilford Press, New York, NY, US

35. Kline R B (2023) Principles and practice of structural equation modeling. The Guilford Press, New York, NY, US

36. Lionetti F, Keijsers L, Dellagiulia A, Pastore M (2016) Evidence of Factorial Validity of Parental Knowledge, Control and Solicitation, and Adolescent Disclosure Scales: When the Ordered Nature of Likert Scales Matters. Front Psychol 7:

37. van de Schoot R, Lugtig P, Hox J (2012) A checklist for testing measurement invariance. Eur J Dev Psychol 9:486–492. https://doi.org/10.1080/17405629.2012.686740

38. Pallant J (2020) SPSS Survival Manual: A step by step guide to data analysis using IBM SPSS, 7th ed. Routledge, New York, NY, US

39. McDonald RP (1999) Test theory: A unified treatment. Lawrence Erlbaum Associates Publishers, Mahwah, NJ, US

40. Anderson D (2022) esvis: Visualization and Estimation of Effect Sizes.

41. Wickham H (2016) ggplot2: Elegant Graphics for Data Analysis. Springer International Publishing, New York, NY, US

42. Wickham H, Averick M, Bryan J, et al (2019) Welcome to the Tidyverse. J Open Source Softw 4:1686. https://doi.org/10.21105/joss.01686
